# A Brief History of Simulation Neuroscience

**DOI:** 10.3389/fninf.2019.00032

**Published:** 2019-05-07

**Authors:** Xue Fan, Henry Markram

**Affiliations:** Blue Brain Project, École Polytechnique Fédérale de Lausanne (EPFL), Geneva, Switzerland

**Keywords:** simulation neuroscience, digital reconstruction, brain modeling, neuronal types, connectome, brain structure and function, history

## Abstract

Our knowledge of the brain has evolved over millennia in philosophical, experimental and theoretical phases. We suggest that the next phase is simulation neuroscience. The main drivers of simulation neuroscience are big data generated at multiple levels of brain organization and the need to integrate these data to trace the causal chain of interactions within and across all these levels. Simulation neuroscience is currently the only methodology for systematically approaching the multiscale brain. In this review, we attempt to reconstruct the deep historical paths leading to simulation neuroscience, from the first observations of the nerve cell to modern efforts to digitally reconstruct and simulate the brain. Neuroscience began with the identification of the neuron as the fundamental unit of brain structure and function and has evolved towards understanding the role of each cell type in the brain, how brain cells are connected to each other, and how the seemingly infinite networks they form give rise to the vast diversity of brain functions. Neuronal mapping is evolving from subjective descriptions of cell types towards objective classes, subclasses and types. Connectivity mapping is evolving from loose topographic maps between brain regions towards dense anatomical and physiological maps of connections between individual genetically distinct neurons. Functional mapping is evolving from psychological and behavioral stereotypes towards a map of behaviors emerging from structural and functional connectomes. We show how industrialization of neuroscience and the resulting large disconnected datasets are generating demand for integrative neuroscience, how the scale of neuronal and connectivity maps is driving digital atlasing and digital reconstruction to piece together the multiple levels of brain organization, and how the complexity of the interactions between molecules, neurons, microcircuits and brain regions is driving brain simulation to understand the interactions in the multiscale brain.

## The Next Phase of Brain Research

Over past millennia, brain research evolved through a series of fundamental transformations of human thinking to approach the mind and the brain. At the dawn of human civilization, mainly based on intuitive and analogical thinking, the deeply philosophical phase relied on subjective experience and “pure reason” (Lamb, 1925), without any empirical method for proving suggested ideas. To gain empirical evidence, mainly based on reductionist thinking, brain research evolved into an experimental phase, by means of observation, measurement and experimentation, which led to the hyper-specialization we see in modern neuroscience. During this phase, huge amounts of disconnected datasets were produced, each limited to a certain level of brain structure and function (Frackowiak and Markram, 2015). To deal with the daunting forests of data, abstraction and simplification methods from physics, mathematics and computer science gave rise to the theoretical phase of neuroscience. This kind of abstractive thinking follows the logic that “if one squeezes out all the complexity from a system, one eventually reaches its essence and then, and then only, does one truly understand the brain.” Theoretical neuroscience tries to interpret experimental data and to gain analytical tractability by simplifying experimental observations, generating concepts and building minimal mathematical models (Gerstner et al., 2012). This phase also gave rise to artificial intelligence and its evolution to its current form today.

Experimental and theoretical phases have developed through three main paths: neuronal mapping that tries to classify and catalog different types of cells in the brain; connectivity mapping that aims to map connectivity between individual neurons (neighboring neurons, neurons in neighboring groups, neurons in distant brain regions), between groups of neurons (layers, columns, nuclei, etc.) and between brain regions (visual area, auditory area, etc.); functional mapping that tries to relate brain function and behavior to the structure of the brain (e.g., role of partial connectomes or the whole connectome).

Neuronal mapping is evolving from subjective descriptions towards objective classifications of cell types, from morphological types (Berlin, 1858; Meynert, 1867; Golgi, 1883; Ramón y Cajal, 1909) to genetic types (Monyer and Markram, 2004; Toledo-Rodriguez et al., 2004; Urban and Rossier, 2012; Wagner et al., 2016) and multidimensional types (e.g., according to a combination of morphological, electrical, afferent, efferent, molecular and genetic types; Markram et al., 2004, 2015; Zeng and Sanes, 2017).

Connectivity mapping is evolving from loose topographic maps of major nerve tracts between brain regions (Vicq-d’Azyr, 1786; Gall and Spurzheim, 1810; Meynert, 1871) towards dense anatomical and physiological maps of connections between individual genetically distinct neurons (Oh et al., 2014; Swanson and Lichtman, 2016). The nomenclature of the types of connections formed in the brain evolves at the pace of the development of the nomenclature of cell types and is set on a path towards a nomenclature for a large addressing system indicating each cell type in the brain.

Functional mapping is evolving from psychological and behavioral stereotypes towards a map of behaviors emerging from structural and functional connectomes (Gall and Spurzheim, 1810; Vogt and Vogt, 1903; Brodmann, 1908; Sporns, 2016), from observing and characterizing brain responses to stimulation (Hitzig and Fritsch, 1870; Penfield and Boldrey, 1937) towards understanding the causal relationship between neural connectivity and brain function (Bassett and Sporns, 2017; Reimann et al., 2017a). Today, at the cellular level, neuroscientists are still surprised to find that different neurons respond to different inputs in a different manner and are still composing an endless spectrum of stimulus preference maps for neurons, while we are moving from considering only how the type of neurons is responsible for their different responses towards identifying the contribution of the underlying networks. At the whole-brain level, studies are beginning to reveal how the underlying connectome shapes, for example, functional magnetic resonance imaging (fMRI) image patterns. At the behavioral level, attempts to map signatures of specific cognitive functions to the underlying structures are still limited to networks of brain regions. As the number of brain regions found to be involved in any cognitive task grows, functional mapping will likely evolve from statistical subgraphs of the brain towards dynamic full graphs.

However, in these three paths, experimental and theoretical approaches are hindered by the barriers of scale and complexity. How can we scale up cellular phenotyping and deal with the dynamics of cellular properties to achieve a comprehensive census of cell types in mammalian brains? How can we rise to the challenge of volume, time and dynamics in full connectome mapping potentially even down to the nanoscale? How can we trace all the molecular and cellular mechanisms that give rise to brain function and behavior?

**Figure 1:**
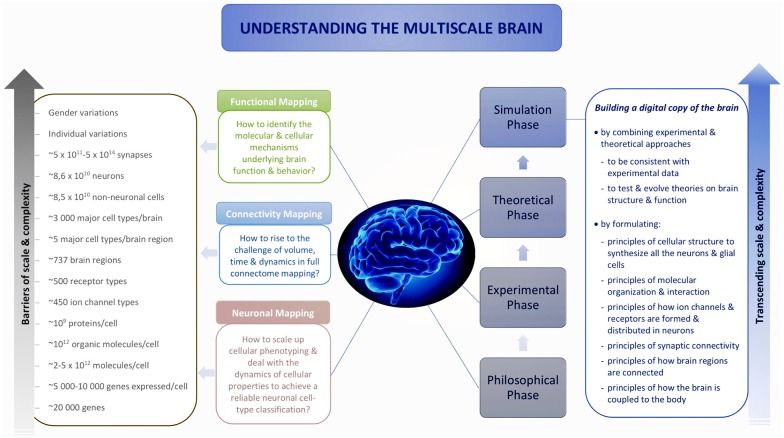
Understanding the multiscale brain.

To transcend these barriers, simulation neuroscience was born. It is arguably the next phase of brain research, after its philosophical, experimental and theoretical phases. Simulation neuroscience combines experimental and theoretical approaches to achieve a dense digital reconstruction of the brain consistent with experimental data, which in itself forms a unifying theory of brain structure and function and which can be used to test and evolve new theories (Figure 1). The goal of simulation neuroscience is to build a digital copy of the brain instead of an arbitrary model, even if that model could imitate certain brain functions (Markram, 2006; Markram et al., 2015). Since neither a comprehensive repertory of data nor a complete map of the brain exists or will likely be obtained purely from experiments, we obviously cannot do this blindly. It requires building the digital copy by formulating principles of cellular structure to synthesize all the neurons and glial cells, principles of molecular organization and interaction, principles of how ion channels and receptors are formed and distributed in neurons, principles of synaptic connectivity, principles of how brain regions are connected, and ultimately, principles of how the brain is coupled to the body. It is through formulating and exercising these principles that simulation neuroscience makes progress systematic and understanding tractable. If correct, these principles allow predicting vast gaps in data and drive a new question: what is the minimal, not maximal, data we need to reconstruct the brain? Indeed, experimental neuroscience should be asking what can be predicted and what must be measured.

Reconstructing the brain recapitulates the history of neuroscience by evolving and accelerating its major steps, from early morphological descriptions of the nerve cell to later electrophysiological and biochemical studies of neural connectivity: synthesize and evolve available knowledge, methods and technologies into a new science, and take a quantum leap onto a path that, in the end, can lead to understanding the multiscale brain. Industrialization of neuroscience and the resulting large disconnected datasets are generating demand for integrative neuroscience; the scale of neuronal and connectivity maps is driving digital atlasing and reconstruction to piece together the multiple levels of brain organization, and the complexity of the interactions between molecules, neurons, microcircuits and brain regions is driving simulation neuroscience to understand the multiscale brain. To explore the origin and making of this paradigm shift, we reconstruct the deep historical paths leading to simulation neuroscience through the philosophical, experimental and theoretical phases of brain research, in particular, from the first observations of the nerve cell to modern attempts to digitally reconstruct and simulate the brain, by identifying the major scientific, technological and conceptual breakthroughs that have guided this passionate quest of humans to understand the brain and their own condition.

## From the Weight of the Heart to Brain Simulation

Humans see and feel, live and die, conscious of their own existence. They think and desire to understand themselves.

About 3,000 years ago, in ancient Egypt, almost 200,000 years after the birth of *Homo sapiens* and 9,000 years after the Agricultural Revolution (Harari, 2014), the heart was still considered to be the seat of emotions and thoughts, weighed after death by gods against a feather representing truth and order to determine the destiny of the deceased: to go to heaven or to be devoured by a monster (“Book of the Dead,” Papyrus of Ani, 1250 BC). The brain, considered trivial, was the first organ to be thrown away during embalming: part of it was drawn out through the nostrils with a crooked piece of iron, and the rest was rinsed with drugs (Herodotus, 1875).

About 2,500 years ago, in Ancient Greece, Alcmaeon of Croton (~460 BC), a great philosopher and pioneer of anatomical dissection, traced the nerves of the sense organs until their terminations in the brain and inferred that the brain was the seat of sensation and thought (Tannery, 1887). Thus were laid the foundations of brain science. One century later, influenced by Alcmaeon of Croton, Plato (~360 BC) located the immortal soul, the *logos* (thinking and reasoning), in the head, since it is in the form of a globe, at the top of the body, close to the heaven, reflecting the perfect image of God and the Universe (Lamb, 1925). The *logos* is a *dæmon* inside each of us, a genius given by God to guide humans to communicate with the divine soul of the Universe. Plato located in the thorax the mortal soul—the *thymos* and the *eros*—our fearful but ineluctable passions and desires. However, in Aristotle’s view (~350 BC), the intellectual soul (*nous*), imperishable and self-existing, which bestowed on humans the ability to understand and which distinguished them from plants and animals, did not operate through any specific bodily organ (Hicks, 1907). Relating sensation to the blood, relying on the idea that the brain was bloodless and cold, Aristotle thought that the heart was the seat of sensation, while the brain was just an organ for cooling the heat produced by the heart (Ogle, 1911).

About 500 years ago, what has become known as the “Scientific Revolution” began (Burtt and Edwin, 1923; Butterfield, 1959). We acknowledged our ignorance (Harari, 2014) and embarked on an exploration of the unknown. Modern science was born. The view of the universe and the nature of human life was transformed through the transition from mainly relying on the internal mind to external observation. To survive and evolve, to increase their capacities and to produce new resources, humans gained knowledge and invented technologies both transmittable to others to accelerate scientific discoveries—therefrom arose the powerful collective scientific process. We explored the world and our own body, including the brain. Brain science accelerated.

About 475 years ago, Vesalius (1543) dissected human corpses, described the anatomy of the brain and first distinguished between gray matter and white matter. About 300 years ago, “fine vessels” were observed within a nerve under a self-made one-lens microscope (van Leeuwenhoek, 1719). More than 100 years later, “large, colorless and free globules” and “granules connected in rows by delicate filaments” were described in leech nervous tissue through an achromatic microscope (Ehrenberg, 1833). About 30 years later, “protoplasmic processes” were identified through carmine staining (Deiters, 1865). In about 150 years, “vessels,” “globules” and “protoplasmic processes” were finally connected together in the human mind to form a single cellular unit, the nerve cell, named later the “neuron” (von Waldeyer-Hartz, 1891).

Where are we now? Our quest to understand the brain has advanced in scale and complexity through the experimental and theoretical phases of brain research. We are beginning to understand the structural and functional diversity of neurons, how they are connected, and how a specific network of neurons gives rise to emergent functions.

However, since Alcmaeon of Croton dissected brains and suggested that the brain was the seat of sensation and thought, almost 2,500 years have elapsed (Tannery, 1887). We still do not understand the basic neural mechanisms underlying brain function, which give rise to our emotions, thoughts and memories (Koch et al., 2016; Südhof, 2017). We remain “strangers to ourselves” (address by Shimon Peres when the Human Brain Project was awarded, the European Parliament, March 12, 2013).

Modern philosophers continue to reason about the mind and the brain in diverse forms. Dualists argue for the irreducibility of conscious experience and sensory qualia—surviving forms of Plato’s and Descartes’ substance dualism. In their view, we will probably never obtain a complete explanation of consciousness based on neural mechanisms—What is it like to be a bat or a zombie (Nagel, 1974; Chalmers, 1996)? Relying on the concept of multiple realizability and the computational theory of mind, functionalists pay little attention to neuroscientific details, presuming that a given mental state can be realized through diverse physical mediums, either a brain or a computer (Fodor, 1975; Putnam, 1965). The rise of neurophilosophy fosters the co-evolutionary research methodology, in particular the co-evolution of philosophy with cognitive and computational neuroscience (Churchland, 1986), with the aim of applying neuroscientific findings to classical philosophical concepts such as morality (Prinz, 2007; Churchland, 2011). On the basis of eliminative materialism, neurophilosophers try to replace the categories of “folk psychology” with neuroscientific ontology (Churchland, 1986). Contrary to dualists, they search for a neurobiological explanation of consciousness, a unified theory of how the mind-brain works (Searle, 1992; Dennett, 1993; Churchland and Churchland, 1997). However, today, this goal still remains vague.

In parallel with these philosophical pursuits, methodologies in neuroscience also evolved by crossing the boundaries between different doctrines and disciplines. Against rationalist Descartes’ “*Cogito ergo sum”* (Descartes, 1905), empiricists argued a half-century later for “*tabula rasa*” and thought that instead of *a priori* reasoning, the nature of the world and the mind could only be understood through empirical research with observations and experimental reasoning (Locke, 1689). This view prepared the philosophical ground for the rise of experimental neuroscience. Influenced by modern mathematical logic developed in the late 19th century (Frege, 1879, 1960), early empiricism further evolved into logical empiricism (Carnap, 1928; Neurath, 1932), which led to the idea of the mind as a logic machine and the computational theory of mind (McCulloch and Pitts, 1943; Putnam, 1965; Fodor, 1975). This gave rise to another phase in brain research—theoretical neuroscience.

Reduction is the major form of reasoning in both experimental and theoretical neuroscience, although it varies from intertheoretic reduction to “reductionism-in-practice” (Hooker, 1981a,b,c; Bickle, 2003). This kind of reasoning has been challenged by several theories of neuroscientific explanations. Causal-mechanistic reasoning aims to capture the unity of neuroscience by producing a mosaic of explanations at different levels, instead of reductive, unifying or model-based forms of scientific explanations (Craver, 2009). However, to the philosophers of neuroscience in search for a unified theory of brain function and behavior, understanding the brain will require both neurobiology and large-scale theoretical frameworks. In this view, a major methodological theme consists in the co-evolution of macrotheory and microtheory, an interanimation of philosophy, psychology, computer science and neuroscience, of top-down and bottom-up research (Churchland, 1986). This endeavor aims to combine multiple disciplines, in particular philosophy and neuroscience, into a unified science, to obtain a unified theory of the mind-brain. However, since the birth of neurophilosophy, more than 30 years have passed, this goal still remains remote. Why cannot we understand the mind-brain?

Brain research over past millennia is like solving a strange jigsaw puzzle that is devoid of a predetermined picture—various pieces have been accumulated semi-randomly in the hope that all the data and knowledge will self-organize. The mind does not have a shape, but the brain does. Instead of imposing arbitrary forms on the mind, can we reconstruct a brain from its basic molecular and cellular units, find out the principles that connect them together and test our theories in a systematic manner? This quest gave rise to simulation neuroscience. What is the philosophy of this new science?

For thousands of years, seeking truth, philosophers have been addressing fundamental questions about the mind and ourselves, yet without producing empirical evidence; their reasoning wanders in the silence of the desert. For hundreds of years, seeking completeness, experimental neuroscientists have been trying to understand every single part of the brain by breaking it into its basic components and have built forests of datasets, but how much more elements are there still to map, are we lost? For nearly a century, seeking a single unified theory, theoretical neuroscientists have been trying to walk out of these forests by cutting down trees; the complexity indeed decreases but also the structural and functional richness of the ecosystem. Finally, have not the models simply become data fitting functions? If several models can fit the data, does it mean that they all explain brain function? To transcend the barriers to these endeavors, can we get an overview of all the forests of datasets, reorganize and integrate them in the context of the whole brain, while filling the gaps that experiments will never be able to fill and finding ways through the forests by considering the ecosystem of the brain? The deep meaning of simulation neuroscience consists in reconstructing and simulating the brain from the most fundamental principles we can isolate to understand and link the multiple layers that form ourselves, from molecules and cells to brain function and behavior, to give meaning and life to data and theories.

**Figure 2:**
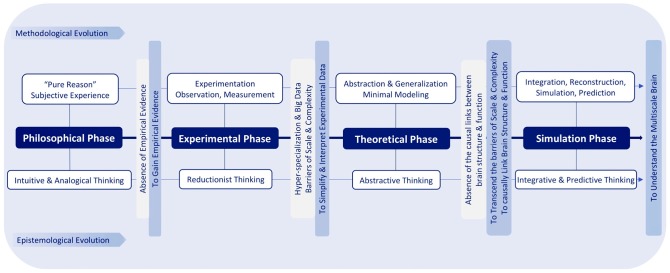
Epistemological and methodological evolution of brain research.

Due to reductionist thinking, experimental neuroscience is hindered by huge amounts of disconnected datasets and seemingly infinite scale and complexity. Based on abstractive thinking, theoretical neuroscience tries to address these problems through simplification but abstracts away detailed brain structures and their emergent functional properties. To reconcile and transcend these two extremes, by leveraging high performance computing, simulation neuroscience approaches the brain through integrative and predictive thinking: integration of experimental and theoretical approaches, integration of disconnected datasets and knowledge and integration of the multiple scales of brain structure and function, in association with predictive methods for filling the gaps (Figure 2).

The brain is a multidimensional network of networks of genes, proteins, cells, synapses and brain regions, all interacting inside a dynamically changing environment of neurochemicals. Brain functions emerge as electrical, chemical and mechanical chain interactions through these networks. Since there is no scientific evidence that we can ignore any kind of these interactions, the only way to understand all aspects of the multiscale brain is to reconstruct and simulate all these types of interactions.

The philosophy of simulation neuroscience originates from the will to transcend the barriers of scale and complexity during the evolution of neuronal mapping, connectivity mapping and functional mapping in the experimental and theoretical phases of brain research. The present review will trace the historical evolution of this pursuit by identifying the major milestones that are the most related to it and that are capable of characterizing it in a concise way, instead of conducting an exhaustive survey of all the investigators whose important work has contributed to the evolution of brain research.

## Neuronal Mapping: From the Birth of the Neuron Towards a Comprehensive Census of Brain Cell Types

Neurons and glial cells constitute the two major cellular populations in the human brain (~86 billion neurons vs. ~85 billion non-neuronal cells; von Bartheld et al., 2016). Although they were probably first described at the same time (Dutrochet, 1824), neurons have been more studied because their electrical excitability correlates well with higher brain functions and are therefore considered essential to brain function and behavior (Galvani, 1791; du Bois-Reymond, 1843). Neurons are divided into diverse types characterized by their morphological, physiological or molecular properties. Just in the retina, the number of neuronal types is estimated to be 100–150, and 2,500–5,000 in the adult mammalian nervous system (Bota et al., 2003; Zeng and Sanes, 2017). Although efforts are underway to try to achieve a comprehensive census of neuronal cell types over the next decade (Zeng and Sanes, 2017), neuronal cell-type classification is controversial and extremely challenging for the future of neuroscience. Even so, then we will still need to ask: “What does each cell type do?”

To better visualize the trajectory of neuronal mapping in the future, we need to understand its origin. What is the history of the neuron, from its first descriptions to modern neuronal classification? First of all, how did humans discover the neuron?

In fact, humans did not discover the neuron; they reconstructed it.

### All Began With a Nerve

About 300 years ago, “fine vessels” were observed within a nerve under a self-made one-lens microscope (van Leeuwenhoek, 1719), clearly identified as axons only more than 60 years later (Fontana, 1781). After about 50 years, “large, colorless and free globules” and “granules connected in rows by delicate filaments” were described in leech nervous tissue through an achromatic microscope, considered yet to be the “excreted nuclei” of red blood cells (Ehrenberg, 1833). Three years later, appeared the first microscopic image of the nerve cell body with the nucleus and nucleolus, but the “primitive fiber” (axon) and the “globule” (soma) were still considered to be separated elements (Valentin, 1836). Nevertheless, in the same year, the anatomical continuity between the nerve fiber and the nerve cell body was observed (Remak, 1836). However, “protoplasmic processes” (dendrites) were only described more than 80 years after the identification of the axon, owing to chromic acid fixation and carmine staining (Deiters, 1865). Only then did humans succeed to reconstruct together the “vessel,” “globule” and “protoplasmic processes” into a single nerve cell, which took almost 150 years.

However, at that time, the soma and fiber of the nerve cell were still considered functionally separated. The nerve cell body, often taken for a trophic center, was thought unnecessary to nerve conduction because most anatomists believed that the nerve fiber ran straight through the cell body (Bernard, 1858; Lorente de Nó, 1935). Therefore, electrophysiology was only based on the study of nerves. Nevertheless, recordings of spinal cord antidromic evoked potentials showed that the polarization of conduction in the spinal cord was not a property of nerve fibers, but rather localized in the soma (Sherrington, 1897). However, it was not until the development of intracellular recording (Hodgkin and Huxley, 1939; Ling and Gerard, 1949), making it possible to characterize local potentials in different parts of a neuron, that the soma and fiber of the neuron were functionally reconstructed together by humans, almost 90 years after the morphological reconstruction of the nerve cell (Eccles, 1952).

Even so, at this stage, humans still did not succeed to completely reconstruct the neuron, hindered by the fierce controversy over the mode of connection between nerve cells. On the one hand, nerve cell anastomotic networks connected by axons and/or by dendrites were observed through ammoniated carmine and gold chloride staining or Camillo Golgi’s “black reaction” (silver nitrate impregnation after fixation with potassium dichromate and osmic acid), which established the reticular theory (von Gerlach, 1872; Golgi, 1875). On the other hand, ontogenetic method and retrograde degeneration method revealed that each nerve fiber originated from a single cell and that the degeneration of the fibers and somas of nerve cells was limited to the units directly affected (Forel, 1887; His, 1887). These observations were later supported by direct histological evidence obtained with improved Golgi’s method, which showed the individuality of each nerve cell and founded the neuron doctrine (Ramón y Cajal, 1888; von Waldeyer-Hartz, 1891).

And yet, neuroscientists at that time were still confronted with another question: how do nerve cells communicate between them? Camillo Golgi thought that the communication between nerve cells and the unified functioning of the nervous system could only be achieved through a continuous network, while Santiago Ramón y Cajal suggested that neural transmission could occur through a kind of “granular cement” or a “particular conductive substance” connecting the surfaces of nerve cells in contact. Ramón y Cajal’s idea announced the concept of the synapse (Foster and Sherringon, 1897), demonstrated later through Loewi’s famous experiment during which a substance collected from a stimulated heart stimulated another heart in the same way as the action of a nerve (Loewi, 1921).

However, it was not until the mid-20th century that the individuality of each nerve cell and the existence of the synaptic cleft were finally confirmed, owing to electron microscopy (EM) observations (Palade and Palay, 1954; De Robertis and Bennett, 1955). Since the first observation of nerve fibers (van Leeuwenhoek, 1719), the human reconstruction of the neuron as an independent cellular unit had taken almost 240 years. How much time would take the classification of different types of neurons?

### A Way Through the “Butterflies of the Soul”

What is the path through the labyrinth of billions of the “butterflies of the soul” (Ramón y Cajal, 1917)? Early researchers first noticed different shapes of nerve cells and named them either by their morphological features or after their discoverers. With the development of histological techniques in the mid-19th century, nerve cells were first classified into pyramidal cells, small and irregular or granular cells and spindle-shaped cells, which founded cytoarchitectonics (Berlin, 1858). Then this morphological classification was further elaborated in association with cortical layers and cell function (Meynert, 1867). About 16 years later, using the “black reaction,” Golgi distinguished two basic types of nerve cells in the cerebral cortex and suggested their functions: Type I cell with a long axon giving off a small number of lateral filaments was motor cell; Type II cell with a short axon divided into many complex lateral branches was sensory cell (Golgi, 1883). However, this functional definition of the two cell types was later refuted by Ramón y Cajal (1894), who observed that Type I cells were abundant in sensory organs and Type II cells were distributed in all nerve centers. This revealed the complex relationship between nerve cell morphology and function. Ramón y Cajal (1909) also attempted to classify neurons by their shapes. However, were these morphological descriptions a reliable way to classify neurons?

Confronted with the subjectivity of these morphological classifications determined by single investigators, some researchers tried to establish objective criteria to classify nerve cells by their electrophysiological or biochemical features. Nissl (1894), using basic aniline dyes, classified nerve cells according to which parts of the cell content were stained and which parts were not and the relationships between the stained and unstained parts. Neurons were also classified by the velocity of their action potentials measured with the cathode ray oscilloscope (Gasser and Erlanger, 1922). Due to a better understanding of the chemical transmission of nerve impulses, neurons were divided into two types: cholinergic and adrenergic cells (Dale, 1933).

However, electrophysiological and biochemical states are limited by their sensitive condition-dependence. Faced with this problem, researchers attempted to characterize neurons with more stable features. With the development of immunohistochemistry in the 1940s and that of RNA and DNA sequencing in the 1970s (Coons et al., 1941; Min Jou et al., 1972; Wu, 1972), molecular classification methods were introduced to classify neurons according to their molecular properties, in particular protein composition and mRNA composition, with the assumption that some molecular features stay permanent to maintain cell identity (Fishell and Heintz, 2013; Deneris and Hobert, 2014). Single-cell transcriptomics, developed in the early 1990s, is considered to have the potential to provide a “complete” census of neuronal types (Toledo-Rodriguez et al., 2004; Poulin et al., 2016; Zeng and Sanes, 2017). High-throughput, multiplexed methods, such as multiplexed fluorescence *in situ* hybridization (FISH) and *in situ* sequencing methods, are being developed to scale up the enterprise of neuronal cell-type classification (Ke et al., 2013; Lee et al., 2014; Chen et al., 2015, 2016).

In 160 years, neuronal classification has evolved from subjective, morphological description to objective, multi-criteria identification; from monothetic approach to polythetic clustering (Berlin, 1858; Ramón y Cajal, 1909; Markram et al., 2004; Migliore and Shepherd, 2005; Armañanzas and Ascoli, 2015). However, a comprehensive census of neuronal cell types is still out of reach. What are the major challenges?

### Towards a Comprehensive Census of Brain Cell Types

Neuroscience aims to achieve a comprehensive census of neurons and glial cells in the brain, with molecular annotation at subcellular resolution, such as mRNA expression, ion channels and synaptic proteins. However, there are ~86 billion neurons in the human brain, and every neuron appears unique; single-cell transcriptome analysis represents only a snapshot due to cyclic and stochastic fluctuations in RNA content (Raj and van Oudenaarden, 2008; Shapiro et al., 2013); gene expression and phenotypic properties of cells can dynamically change in response to internal and external cues (Cohen and Greenberg, 2008; West and Greenberg, 2011). Due to these factors, all neuronal classifications are provisional and hypothetical.

Faced with these challenges, how can we build a way through billions of the “butterflies of the soul”? It is true that there are ~86 billion neurons in the human brain, but it is possible to define a minimum sample size able to reliably reveal distinct types. It is true that every neuron appears unique, but we have to reduce dimensionality by defining a relevant level of granularity to identify neuronal types. It is true that gene expression in cells is dynamic, but we have to find out their molecular ground states that maintain cell identity. So, the question is: how can we overcome the barriers of scale and complexity to achieve a reliable neuronal cell-type classification?

## Connectivity Mapping: From White Matter Tracts Towards a Full Connectome

### Leaves of a Cabbage

Arising from a stem, dispersed into leaves spreading out in a circular shape to form cavities, in the eyes of a 17th-century anatomist, the extending nerve tracts in the brain formed loose nets and ventricles like the leaves of a cabbage (Malpighi and Fracassati, 1669). Since Ancient Greece, nerve tracts had been considered related to brain function (Tannery, 1887). A question then arose: how to trace these tracts?

About 330 years ago, white matter was observed to be composed of fibrils arranged in bundles through the scraping method of dissection (Vieussens, 1684). A century later, nerve tracts were divided into inter- and intra-hemispherical pathways (callosal and association systems; Vicq-d’Azyr, 1786). The first category connected the two hemispheres, including the corpus callosum, the corpora quadrigemina, the anterior and posterior commissures, the cerebral peduncles, the pons, the anterior medullary velum, the interthalamic adhesion and the trigeminal tubercle. The second category was supposed to assure the communication between the base and other parts of the brain, including the arcuate fasciculus, the pillars of the fornix, the peduncles of the pineal gland, the tracts connecting the mammillary tubercles and the anterior thalamic tubercles. More than 20 years later, the projection system was identified through blunt dissection, including afferent and efferent fiber pathways linking the cortex with the subcortical regions, the brain stem and the spinal cord (Gall and Spurzheim, 1810).

However, dissection techniques could not determine the precise trajectory and arrangement of nerve tracts. Detailed tract tracing only became possible with the development of histological methods. Using a Zeiss-microscope and carmine or gold chloride staining, Theodor Meynert identified clearly the three main types of white matter tracts: the association systems—the short arcuate fibers and long association fibers connecting the various parts of the cerebral cortex; the commissural pathways connecting the two hemispheres; the afferent and efferent projection systems linking the cortex to the subcortical structures (Meynert, 1871).

Early tracing studies, relying on physical diffusion of dyes in fixed material, were limited to large fiber tracts between brain regions. The studies of neurocircuitry required more refined methods applicable to living tissue. Degeneration methods inferred neuronal connectivity from pathological changes following experimental lesions to the nervous system (Türck, 1849; Waller, 1850; von Gudden, 1870; von Monakow, 1897). However, lesions were usually nonspecific, degeneration altered the normal morphology of neurons, and pathological changes were extremely variable (Cowan et al., 1972).

To remedy this, tracing methods exploiting axonal transport in living neurons were developed in the 1970s. Retrograde tracing techniques introduced an enzyme or fluorescent tracer in a downstream location relative to the targeted neurons, capable of labeling the somas of the neurons projecting to the injection site, but unable to visualize the fiber pathways linking them (Kristensson, 1970; Kristensson and Olsson, 1971; LaVail and LaVail, 1972). This problem was resolved by anterograde tracing techniques, based on macromolecule transport from the soma to the axon terminals, such as autoradiographic tracing method (Cowan et al., 1972).

Nevertheless, injections of tracers usually resulted in indiscriminate labeling of different types of neurons, and the surgical procedure to introduce an exogenous tracer was complex. To deal with this, tracing techniques exploiting genetic engineering were developed more than 20 years ago (Prasher et al., 1992; Chalfie et al., 1994), which use intrinsic fluorescence to label exclusively the projections of neurons that express a specific molecular phenotype (Feng et al., 2000; Livet et al., 2007; Kuhlman and Huang, 2008). These techniques were even adapted for live imaging of intact animals such as *Drosophila* (Boulina et al., 2013). The leaves of a cabbage have become a forest of rainbow trees.

However, these tracing methods are limited to anatomical connectivity, which alone is not sufficient to account for brain function, because the synapse is dynamic (Tsodyks and Markram, 1997). Therefore, physiological methods were invented. Owing to intracellular recording techniques, synaptic plasticity was better understood, such as the quantal release of neurotransmitters (Fatt and Katz, 1952), central synaptic inhibition (Coombs et al., 1953), short-term synaptic plasticity (Curtis and Eccles, 1960) and spike-timing-dependent plasticity (STDP; Markram and Sakmann, 1995). Neural plasticity also inspired theoretical studies, such as Hebbian cell assembly and learning rule (Hebb, 1949) and the theoretical study of STDP (Abbott and Blum, 1996; Gerstner et al., 1996). Theoretical approach abstracts away detailed biological mechanisms to loosely model neural connectivity by building artificial neural networks. About 75 years ago, the first mathematical model of a simplified neural network appeared (McCulloch and Pitts, 1943), which led to the computational theory of mind and machine learning. This model then evolved into more sophisticated ones, in particular, multilayer perceptrons (Rosenblatt, 1957), recurrent neural networks (Hopfield, 1982) and convolutional neural networks (Cireşan et al., 2011). However, to get deep insights into the detailed neural structures and mechanisms underlying brain function, we still need biologically realistic models.

Although the aforementioned experimental methods are able to trace neuronal connections on the cellular or even molecular scale, these invasive techniques are limited to postmortem brain tissue and experimental animals. To better understand our own brain, would it be possible to trace the neural connections in the living human brain? In the early 1970s, the development of noninvasive neuroimaging techniques, in particular MRI, made it possible to study the structural and functional connectivity of the human brain *in vivo* (Damadian, 1971; Lauterbur, 1973). Nowadays, human brain atlasing combines MRI with gene expression studies, such as the Allen Human Brain Atlas that comprises a comprehensive “all genes–all structures” array-based dataset (Shen et al., 2012). Nevertheless, generally, MRI methods can only trace neural connections between brain regions usually with millimeter resolution.

Over the past 300 years, connectivity mapping has evolved from gross tracing of major tracts in fixed brains to mapping neuronal projections with cellular and molecular resolution in living tissue, from mapping static neural connectivity to dynamic synaptic plasticity, from postmortem studies to *in vivo* large-scale mapping of human brain connectivity including structure, function and gene expression. Is it possible to experimentally map all the neural connections of the brain—the “connectome”?

### Towards Completeness

Science dreams of completeness. Since the emergence of the term “genome” in 1920 (Winkler, 1920), fostered by technological advances in large-scale, high-throughput research, the “ome” has become a doctrine, aiming to capture all the parts of biological systems and their interactions (Sporns, 2013b). Inspired by the “genome,” the term “connectome” was introduced in 2005, initially referring to a comprehensive structural description of the network of brain elements and connections (Sporns et al., 2005) or the set of all neuronal connections of the brain (Hagmann, 2005). “Connectomics” aims to map the connectome on the macro-, meso-, micro- and nano-scales and to explain its relation to brain functions (Hagmann, 2005; Sporns, 2013a; Swanson and Lichtman, 2016).

The concept of the connectome originated from the long-held belief that neural connections are related to brain functions, as illustrated by tract tracing since the 17th century. This relationship has been further revealed by recent research: at the microscale, synaptic connectivity is linked to neuronal network dynamics (Chambers and MacLean, 2016); at the macroscale, the anatomical connectivity of the brain is related to its functional connectivity and different states (Hermundstad et al., 2014), and the “connectivity fingerprint” of brain regions may predict their specific functional properties (Saygin et al., 2016; Tavor et al., 2016).

Since the function of neural circuits and systems cannot be explained only through wiring diagrams, we also need information such as the types of neurons and synapses, the dynamics of neuronal synchronization, and the role of different types of glial cells and neuromodulators (Sporns, 2013b; Fields et al., 2015). Therefore, the concept of the “connectome” is evolving to include all the structural and functional relationships between different types of neurons, as well as all their connections with their cellular partners in a defined neural region or the whole brain (Marc et al., 2013; Sporns, 2016; Swanson and Lichtman, 2016). Nevertheless, this concept owes its origins to MRI methods, which enable *in vivo* rapid-throughput mapping of human brain connectivity at the macroscale.

#### Mapping Long-Range Neural Connections Between Gray Matter Regions

Macroconnectomics aims to map all the neural connections between gray matter regions at millimeter resolution. It is best suited to *in vivo* human studies with neuroimaging methods, where few of fine-scale methods used in laboratory animals are applicable (Sporns, 2013b; Van Essen, 2013). MRI, the major noninvasive neuroimaging technique for *in vivo* human connectome mapping, was developed in the early 1970s, first used to diagnose cancer (Damadian, 1971; Weisman et al., 1972; Lauterbur, 1973). Described as “*in vivo* Brodmann mapping” (Brodmann, 1908; Turner and Geyer, 2014), MRI cerebral cartography has inherited the long tradition of connectivity mapping, established since the 18th century (Vicq-d’Azyr, 1786).

Diffusion MRI (dMRI) is the main MRI method of mapping structural connections of the brain (Glasser and Van Essen, 2011; Craddock et al., 2013). Invented in the 1980s, dMRI uses water diffusion anisotropy along myelinated axons to map large white matter fiber bundles, combined with probabilistic tractography to estimate fiber trajectories (Le Bihan and Breton, 1985; Margulies et al., 2013; Le Bihan and Iima, 2015). About 30 years ago, the first dMRI images of the human brain were obtained at 0.5T, with an in-plane spatial resolution of 1.09 × 1.09 mm (Le Bihan et al., 1986). Since then, the sensitivity to diffusion has augmented about 100 times (McNab et al., 2013). To improve spatial resolution of white matter fiber tracking, ultrahigh field magnetic resonance engineering is a basic solution. MRI for clinical use is usually at 1.5T or 3T, and more recently at 7T. The first human 8T MRI was installed in 1999 (Robitaille et al., 1999), and 18 years later, a human whole-body 11.7T MRI (Quettier et al., 2017). Efforts are underway for human 14–20T MRI (Ekosi 20 Tesla Project, 2018). Human brain *in vivo* imaging was already performed at 9.4T (Vaughan et al., 2006); rodent brain and human postmortem tissue imaging at 21.1T (Qian et al., 2012). The final resolution also depends on the acquisition and reconstruction of diffusion images. For example, reconstructing nerve fiber orientations, especially in brain regions where fibers of multiple orientations intersect, involves a trade-off between the accuracy of the peak orientation and the sensitivity to crossing fibers and minor fiber bundles (Van Essen et al., 2012; Lowe et al., 2016). Hitherto, the highest resolution for the human brain achieved at 7T is 0.2 mm, owing to motion correction methods (Stucht et al., 2015). However, even this rare performance is not sufficient to study the connections between individual neurons.

Functional MRI (fMRI) is the main MRI method for studying functional connections in the human brain. Developed in the early 1990s, fMRI first used contrast agents administrated intravenously (Belliveau et al., 1991), then exploited correlations in blood oxygen level dependent (BOLD) signals, based on different magnetic susceptibilities of oxygenated and deoxygenated hemoglobin to detect functional correlations between brain regions (Ogawa et al., 1990, 1992; Bandettini et al., 1992; Kwong et al., 1992). Functional MRI includes two main methods: resting-state fMRI (rsfMRI), measuring correlations in spontaneous activity between brain regions in resting subjects, and task-evoked fMRI (tfMRI), trying to detect functionally distinct brain regions during various tasks such as visuomotor or cognitive processes (Glasser et al., 2016). Almost 30 years ago, human fMRI studies were mostly performed at 1.5T with a spatial resolution of 3–4 mm (Bandettini et al., 1992; Kwong et al., 1992). Since then, the spatial resolution of fMRI has been largely improved, such as the achievement of 0.65-mm resolution in the human brain at 7T (Heidemann et al., 2012), but this is still not sufficient to study how individual neurons are connected to generate brain functions. Furthermore, the temporal resolution of fMRI is fundamentally limited by the nature of BOLD signals, which only indirectly reflect neuronal activity. Due to the temporal dynamics of neurovascular coupling, the peak of BOLD response to a neural stimulus occurs with 5–6 s delay (Glover, 2011). Finally, as a result of artifacts and noises, neurobiologically relevant signals represent only ~4% of primary data (Glasser et al., 2013).

Although MRI is a useful tool for studying human brain connectivity *in vivo*, it offers little data on the connectivity between neurocircuits and between individual neurons that is essential for understanding the mechanisms underlying brain function. Hence the need for meso-, micro- and nano-connectomics.

#### Mapping Connections Between Neuronal Groups and Between Individual Neurons

Meso- and micro-connectomics aim to map all the connections between different neuronal groups defined by cell types or connectivity patterns and between individual neurons at the micrometer scale. Such studies, using invasive techniques, are limited to experimental animals and postmortem human brain tissue. The first mesoconnectome, capturing cell type-specific connections as well as short- and long-range interregional axonal projections, was achieved in the mouse in 2014, through enhanced green fluorescent protein (EGFP)-expressing adeno-associated viral vectors and high-throughput serial two-photon tomography (Oh et al., 2014).

Single-cell staining is the first and most influential method for studying neural circuits at microscale, established by Golgi and Ramón y Cajal in the late 19th century (Golgi, 1875; Ramón y Cajal, 1888). However, dyes could only be applied to small blocks of tissue, making this method unsuitable for tracing long-distance connections. To resolve this problem, chemical markers were injected into circumscribed neural areas, which, however, could not label selectively different types of neurons (Kristensson, 1970; Kristensson and Olsson, 1971; Cowan et al., 1972; LaVail and LaVail, 1972). This was later remedied by transgenic multicolor labeling strategies such as “Brainbow” (Livet et al., 2007). More recently, non-optical, high-throughput methods were invented, such as Barcoding of Individual Neuronal Connections (BOINC), which barcodes individual neurons and introduces transsynaptic viruses to map synaptic connections, based on high-throughput DNA sequencing (Zador et al., 2012). Nevertheless, due to several factors, connectivity reconstructed by this method is difficult to interpret as neuronal connectivity with single-synapse precision (Oyibo et al., 2018).

Light microscopy was, at the origin of the history of neuroscience, the major tool for elucidating the problem of intra-/inter-neuronal and interregional connectivity in the brain. However, conventional light microscopes cannot resolve neural structures smaller than ~0.25 μm, due to the diffraction barrier identified almost 150 years ago (Abbe, 1873). This barrier was finally broken by super-resolution microscopy developed in the late 20th century, which can routinely resolve a few tens of nanometers, such as stimulated emission-depletion (STED) microscope (Hell and Wichmann, 1994), structured illumination microscopy (SIM; Gustafsson, 2000) and photoactivated localization microscopy (PALM; Betzig et al., 2006). Yet, even so, major challenges still lie ahead, in particular, mapping connections in small neural areas where many cells are targeted at the same time and where the connection density is high (Lichtman et al., 2008). This may require a resolution of a few nanometers (Huang et al., 2010). How to map neuronal connections at this scale?

#### Mapping Neural Connections at Individual Synapses and Gap Junctions

Nanoconnectomics uses EM, the only method capable of identifying unequivocally synapses and gap junctions at nanometer or even sub-nanometer resolution. EM provides high-resolution validation of macro-, meso- and micro-connectomes.

The first electron microscope, a transmission electron microscope (TEM), was built in 1931, only capable of 14.4× magnification (Ruska, 1993). However, 2 years later, the resolving power of the TEM (12,000×) surpassed already the resolution limit of light microscopy at that time (Ruska, 1993). Another major type of EM is scanning electron microscopy (SEM), introduced in 1937 (von Ardenne, 1937), capable of sub-nanometer resolution (Masters et al., 2015). TEM remains to date the highest resolution technology able to validate specific gap junctions and small synapses requiring, for example, 0.3 nm resolution (Marc et al., 2013). Recently, using the aberration correction technique, scanning TEM (STEM) has even achieved a sub-ångström resolution of 45 pm (Sawada et al., 2015).

However, EM methods are extremely time-consuming and labor-intensive, so currently limited to very small postmortem specimens. The first and the only almost complete nanoconnectome, that of *Caenorhabditis elegans* hermaphrodite, whose nervous system has in total 302 neurons, was achieved in 1986 with serial-section TEM, containing about 5,000 chemical synapses, 2,000 neuromuscular junctions and 600 gap junctions (White et al., 1986). Today, studies continue to fill the gaps in this original connectome and to address further questions such as the nature of individuality and how genetic and environmental factors regulate connectivity (Mulcahy et al., 2018).

The goal of connectomics is to experimentally map a full connectome of the mammalian brain, and ultimately the human brain. Is this achievable?

### Metaphor and Myth

#### What About Biological Reality?

Although MRI methods are capable of large-scale, rapid-throughput mapping of human brain connectivity at macroscale, MRI-derived macroconnectomes result from data reduction, simplification and assumptions, and they do not necessarily reflect the actual structure and function of the brain. They are even described as “metaphors” or “caricatures” (Catani et al., 2013; Margulies et al., 2013).

MRI methods suffer from low spatial resolution. The isotropic voxel size often used is 2 mm (dMRI) or 3 mm (rsfMRI) at 3T and 1–2 mm at 7T. However, the human cerebral cortex contains on average 40,000 neurons and 3 × 10^8^ synapses/mm, and the white matter contains ~300,000 axons/mm^2^ (Van Essen et al., 2012).

The fundamental concept of dMRI consists in using water molecules to probe neural tissue structure (Le Bihan and Johansen-Berg, 2012). However, the basic mechanism underlying water diffusion in neural tissue, especially the role of cell membranes in modulating water diffusion, remains to be clarified, hence the fundamental limitation of the sensitivity of dMRI resides in the complexity of water diffusion in the microenvironment of the brain (Van Essen et al., 2014; Le Bihan and Iima, 2015). MRI tractography is indirect and probabilistic: it reconstructs neuronal connections by estimating the “most likely” fiber orientations at every voxel, which may contain tens of thousands of diverging axons; it produces more invalid than valid bundles (Margulies et al., 2013; Maier-Hein et al., 2017). MRI tractography is also biased towards some brain regions, such as the famous “gyral bias,” induced by current fiber tracking algorithms which tend to track towards gyral crowns rather than the walls of sulci or the sulcal fundi (Van Essen et al., 2014; Schilling et al., 2018). The signal-to-noise ratio (SNR) in subcortical regions is usually weaker than in cortical regions, mainly due to their buried location relative to the head coil (Uğurbil et al., 2013). Data processing introduces artifacts and distortions that are difficult to distinguish from actual neural connections (Jones et al., 2013).

The sensitivity of fMRI is affected by the fundamental problem of neurovascular coupling. BOLD signals reflect a complex combination of vascular system dynamics as well as the activity of neurons, astrocytes (Iadecola and Nedergaard, 2007), interneurons (Cauli et al., 2004), pericytes (Hall et al., 2014), vascular endothelium (Hillman, 2014) and smooth muscle cells (Cipolla, 2009). However, the way all these elements contribute to fMRI signals still remains to be clarified. Furthermore, fMRI detects only functional correlations between brain regions, and most functional connections show significant temporal fluctuations depending on measurement and analysis methods—they do not necessarily reflect the causal relationships between neural connections (Friston, 2011). This means that the interpretation of results is often doubtful.

From this point of view, current macroconnectome maps do not offer an actual image of the brain. Reproducibility is also a major concern for MRI studies (Zuo et al., 2014).

#### Volume, Time and Dynamics

The major challenge for micro- and nano-connectomics is the huge number of neurons in the human brain: ~86 billion (Herculano-Houzel, 2012). With current techniques, it would take ~10 million years to map all the synapses in a single human brain (Morgan and Lichtman, 2013). Moreover, the reconstruction of a complete nanoconnectome would only be possible in some invertebrates or simple nervous systems, because the magnification required to visualize synapses produces very small images of tens of μm^2^ (DeFelipe, 2015).

The storage and processing of gigantic volumes of data are problematic (Schreiner et al., 2017). The first fairly complete reconstruction of the *C. elegans* nanoconnectome required ~10,000 EM images (White et al., 1986). Recent local circuit mapping by EM has high data output rates of gigabytes per minute (Helmstaedter and Mitra, 2012). At synaptic resolution, a human brain may require ~2 million petabytes (Swanson and Lichtman, 2016). And this is just for the anatomical data, but what if we include the electrophysiological, biophysical and biochemical counterparts?

Although section preparation automation techniques such as SBF (serial block-face) SEM (Denk and Horstmann, 2004) and ATUM (automated tape ultramicrotomy) SEM (Schalek et al., 2011) were invented and data acquisition has been accelerated through parallel image processing (Eberle et al., 2015), the automation of large-scale image segmentation and reconstruction remains the fundamental bottleneck for EM. Methods such as machine learning and crowdsourcing are gradually reducing the problem (Kim et al., 2014; Greene et al., 2016; Staffler et al., 2017), but no existing computational segmentation algorithm is accurate enough to completely replace human annotators. A recent reconstruction of the nanoconnectome of 950 neurons in the mouse retina took ~30,000 h (Helmstaedter et al., 2013). At current speeds, the complete reconstruction of the nanoconnectome of the human brain may require ~14G person-years (Plaza et al., 2014).

Therefore, it seems impossible that we will ever resolve the full micro- or nano-connectome of any mammal by only relying on experimental methods (Schröter et al., 2017), which in the opinion of many researchers, is nothing more than a myth (Catani et al., 2013). Moreover, the very concept of “full” connectome mapping is problematic: (1) due to connectivity deduction from primary experimental data, individual variability and the parallel application of multiple imaging, reconstruction and analysis methods, any unified map would be based on probabilistic representations of connectivity data (Sporns, 2013b); (2) all the molecular and cellular components of the nervous system are constantly resynthesized or replaced; development involves changes in myelination and the number of neurons; synaptic connections are subject to continuous rewiring and changes in strength and dynamics driven by experiences (Markram and Tsodyks, 1996; Holtmaat and Svoboda, 2009; Bennett et al., 2018; Roelfsema and Holtmaat, 2018). Therefore, any connectome map represents only a snapshot of the dynamic brain; and (3) neurons can rapidly change their functional roles in response to chemical signals such as peptides, hormones or neuromodulators, all with no visible modification to the connectivity diagram, and each wiring diagram can encode many possible circuit outcomes (Bargmann and Marder, 2013).

However, if we want to understand the neural mechanisms underlying brain function, we have to identify their constituent neural connections from the molecular and cellular levels to the whole brain. Facing the “metaphor” of macro-connectomics and the “myth” of micro- and nano-connectomics, how can we overcome the barriers of scale and complexity to reconstruct the neural connections that give rise to brain function?

## Functional Mapping: From Cranial Bumps Towards Neural Mechanisms

### Feeling the Bumps of the Skull

What is the link between verbal memory and bulging eyes, the cerebellum and sexuality? About 200 years ago, early attempts to localize brain functions and behaviors in cerebral structures began with Franz Gall’s phrenology (Gall and Spurzheim, 1810). The brain was considered to be composed of different “organs,” each with its own function, and the size of cortical organs depended on the development of mental faculties, reflected through cranial bumps. Gall noticed that individuals with a retentive verbal memory had bulging eyes and that several cases of aphasia were caused by the damage to the frontal lobe. Therefore, he localized verbal memory in the frontal lobes, assuming that the super development of these lobes pushed out the eyes. Feeling the burning nape of a nymphomaniac widow, he considered the cerebellum to be the organ of the sexual instinct (Gall et al., 1838). Although phrenology was based on such false assumptions, it drove the functional mapping of the brain. After all, Gall was not completely wrong with the relation between brain structure and function, which has been partly supported by some modern studies, in particular, the famous MRI study showing that London taxi drivers have larger posterior hippocampi (Maguire et al., 2000).

To surpass the simplistic correlation between cranial bumps and mental faculties, functional mapping further developed in cytoarchitectonics and myeloarchitectonics to build maps of cerebral regions according to their structure and inferred function. Motor function was one of the first functions to be located in the brain, owing to the identification of the giant pyramidal cells (Meynert, 1867; Betz, 1874; Lewis, 1878; Campbell, 1903). Cécile and Oskar Vogt mapped 200 structural and functional areas in the monkey cortex, using myelin-stained histological sections (Vogt and Vogt, 1903). Five years later, Brodmann (1908) distinguished 43 cytoarchitectonic areas in the human cortex, using cell body-stained histological sections, and assigned to each of them a function. Although today Brodmann’s map is still largely used to localize neuroimaging data (Turner and Geyer, 2014), it does not match recent anatomical and functional data in many brain regions, and the mosaic-like segregation of the cerebral cortex is far from reflecting its heterogeneous structure (Amunts and Zilles, 2015).

Methods in cytoarchitectonics and myeloarchitectonics mapped brain functions to brain areas mainly by inference. To relate directly behaviors to brain regions, clinicopathological correlation was one of the first methods developed in the history of functional mapping. The faculty of speech was located in the anterior lobes, the lesions to which led frequently to the loss of speech (Bouillaud, 1825; Broca, 1861). Motor centers were located in the region of the middle cerebral artery through the observation of “Jacksonian seizures” with unilateral convulsions (Jackson, 1870). These early studies suggested that the brain consisted of specific, circumscribed, yet interconnected functional areas, the disconnection of which caused neurological disorders. This led to the concept of disconnection syndromes, caused by the destruction of either the centers of convergence where crucial associations were formed or the conduction pathways transmitting information between these centers (Wernicke, 1874; Dejerine, 1892). The concept of disconnection syndromes was further developed in the 1960s: the studies of split-brain patients revealed the topographic organization and functional specificity of the corpus callosum (Gazzaniga et al., 1962), and neo-associationism reinterpreted apraxia, amnesia, agnosia and hemispatial neglect (Geschwind, 1965a,b). However, the phenomenon of “diaschisis” questioned localization of brain functions: the destruction of a cortical area could produce transient symptoms in other distant areas, which showed that immediate symptoms were not a reliable guide to the function of a destroyed cortical area (von Monakow, 1914). This was one strong argument held by holists. They considered that brain functions were distributed continuously throughout the brain: stimulation of a single point in the nervous system stimulated the whole system; a weakened point weakened the whole system (Flourens, 1842). In the late 20th century, brain functions and dysfunctions were further investigated *in vivo* in human subjects with neuroimaging techniques, in particular positron emission tomography (PET) and fMRI (Frackowiak, 1986, 1994). Today, the relationship between segregation and integration, localized and distributed aspects of brain functions still poses a major challenge to neuroscience (Cauda et al., 2014; Sporns, 2014), and new approaches are mandatory (Frackowiak and Markram, 2015).

To directly test the function of brain regions, experimental methods, in particular, electrical stimulation and ablation techniques were developed. Through electrical stimulation that induced motor responses, the motor centers were first mapped in the dog cerebral cortex (Hitzig and Fritsch, 1870), then in a patient with a cranial malformation exposing parts of both cerebral hemispheres (Bartholow, 1874). These results were demonstrated by destructing the motor centers in the monkey brain, which caused motor paralysis totally dissociated from sensory paralysis (Ferrier, 1875). However, the localization of the motor centers was questioned by the “functional instability” of the motor cortex, revealed by stimulating repetitively the same point in the motor cortex (Brown et al., 1912), which suggested that the motor cortex was a changing organ. The famous “sensory and motor homunculi” were built through electrical stimulation of the cerebral cortex in conscious patients undergoing surgery for epilepsy (Penfield and Boldrey, 1937). Owing to ablation techniques, vision was located in the occipital lobe and auditory function in the temporal lobe (Panizza, 1855; Munk, 1890). And ablation of the frontal lobe in monkey was found to disintegrate the personality and to destroy the ability to classify and synthesize groups of representations (Bianchi, 1920). However, these experimental methods suffered from low resolution and lacked specificity.

With the development of single-cell recording techniques, in particular tungsten microelectrodes invented in the 1950s (Hubel, 1957), specific brain functions were localized in certain populations of cells, such as “complex cells” in the visual cortex with specific oriented receptive fields (Hubel and Wiesel, 1962), “place cells” in the hippocampus that respond to stimuli in specific spatial locations (O’Keefe and Dostrovsky, 1971), “face cells” in the superior temporal sulcus that respond selectively to faces (Desimone et al., 1984), and “mirror neurons” in the rostral part of the inferior premotor cortex that become active not only during the execution but also during the observation of specific movements (di Pellegrino et al., 1992). During the same period, theoretical neuroscience explored brain functions through mathematical modeling, such as Marr’s famous models of visual processing widely adopted in computer vision (Marr, 1982). Nevertheless, both of these approaches could not resolve how different types of brain cells and circuits interact together to generate the full array of diverse brain functions.

Over the past 200 years, functional mapping has developed from correlation-based methods to experimental perturbation of brain activity; from observing correlations between cranial bumps and behavioral stereotypes, cyto-/myelo-architectures and brain functions, brain lesions and behavioral deficits, to relating brain regions to behavioral outputs through electrical stimulation or ablation techniques; from localization of brain functions in brain regions to those in specific populations of cells. Functional mapping is evolving towards causally linking brain structure to function with high resolution and specificity. How does modern neuroscience face this major challenge?

### Recording and Manipulating Neural Activity

Current correlation-based methods are particularly represented by fMRI studies that detect the similarity of regional activation profiles reflected indirectly in BOLD signals to extract patterns of correlation or covariance and to infer functional connectivity between brain regions. Trying to correlate neural connections and brain regions to pre-defined behavioral categories, this approach is described by some researchers as “neophrenology” (Miller, 2008). The biophysics of how BOLD signals relate to underlying neural activity remains an unsolved question and represents a fundamental limitation of fMRI studies (Hillman, 2014; Gao et al., 2017). Since correlation-based methods deliver non-causal similarity-based metrics of statistical dependence (Bassett and Sporns, 2017), other methods are used to unravel the causal relationship between neural activity and brain function, in particular recording and manipulating neural activity and observing the behavioral outputs.

About 150 years ago, resting and action potentials were first recorded from frog sciatic nerves with a differential rheotome (Bernstein, 1868). Almost 80 years ago, the first intracellular recording of individual neurons was achieved in the squid giant axon with glass microelectrodes (Hodgkin and Huxley, 1939). Ten years later, voltage clamp was developed, and patch clamp in the 1970s (Cole, 1949; Marmont, 1949; Neher and Sakmann, 1976). About 60 years ago, implantable microelectrodes were developed to record from single neurons in a freely behaving ground squirrel during 4 days (Strumwasser, 1958). Nowadays, penetrating multi-electrode arrays (MEAs) can record from individual neurons simultaneously at multiple sites to study distributed neural circuits (Gehring et al., 2015; Maccione et al., 2015), and mesh nanoelectronics, which are tissue-like electronics consisting of a macroporous mesh structure with addressable electronic devices, have achieved stable single-neuron level chronic recording and stimulation in freely behaving animals for at least 8 months (Fu et al., 2016). Yet, even so, the huge number of neurons and the complexity of neural interactions preclude the high-density parallel recordings of the whole mammalian brain.

Almost 230 years ago, experimental manipulation of neural activity began with electrical stimulation of nerves. The first electrophysiological experiments were achieved in frog neuromuscular preparations through electrical stimulation of sciatic nerves by using electric machine or atmospheric electricity during lightening (Galvani, 1791). Electrical stimulation provides high temporal resolution and can be used in humans to modulate neural activity, such as deep brain stimulation, introduced in clinical practice in the 1950s to treat psychiatric disorders such as schizophrenia (Delgado et al., 1952) and neurological disorders such as Parkinson’s disease (Benabid et al., 1987). Multi-electrode arrays were developed in the 1950s to record and manipulate neural activity in living laboratory animals (Strumwasser, 1958) and are evolving towards chronic, large-scale recording and stimulation at the single-neuron level in freely behaving animals (Fu et al., 2016). Optogenetics, developed in the early 21st century, has been generalized during the last decade to test and generate hypotheses on brain function in non-human neuroscience, using genetically encoded light-activated proteins to manipulate cell activity with cell type-specific and high temporal resolution (Zemelman et al., 2002; Boyden et al., 2005; Lima and Miesenböck, 2005). Nevertheless, it is extremely challenging to control separately all of the cells in the mammalian brain with high spatiotemporal resolution during behavior, particularly due to light scattering and power deposition requirements (Deisseroth, 2015).

Noninvasive approaches such as EEG and MEG are suitable for human studies and long-term monitoring of brain activity, but their low spatial resolution precludes studies at the cellular level (Babiloni et al., 2009; Wendel et al., 2009). Efforts are underway to measure at the cellular level brain activity in persons carrying recording or stimulation electrodes or neurotechnological devices for therapeutic applications or experimental studies, such as deep brain stimulation and brain-machine interface (Moran, 2010; Lozano and Lipsman, 2013). However, these studies are not scalable to large-scale monitoring. Noninvasive stimulation techniques for human studies usually activate brain areas on a centimeter scale, such as transcranial magnetic stimulation, introduced in 1985 to stimulate the human motor cortex for neurological examination (Barker et al., 1985). These techniques lack accuracy and specificity.

Over the past 200 years, experimental studies trying to unravel the causal relationship between neural activity and behavior have evolved from recording and stimulating nerves in frog neuromuscular preparations to chronic monitoring and manipulation of individual neurons in freely behaving animals, from electrical stimulation and ablation techniques to optogenetic manipulation with cell type-specific and high temporal resolution, from univariate correlation between brain regions and behavioral stereotypes to large-scale multivariate monitoring and manipulation of neural circuits, with the ultimate goal of producing a dense functional map of the dynamic brain (Insel et al., 2013).

However, to demonstrate the causal relationship between neural activity and brain function, dense functional mapping requires in principle a comprehensive map of the connectome and the parallel recording from the interacting molecules, cells, circuits and areas throughout the brain. Even with technological advances, dense functional mapping of the whole brain is extremely challenging and thus considered by many researchers to be science fiction (Shen, 2013). How can we overcome this challenge to identify all the molecular and cellular mechanisms underlying brain function and behavior?

### Identifying the Molecular and Cellular Mechanisms Underlying Brain Function and Behavior

Quantifying behavior is a major challenge to studies that aim to identify the neural correlates of pre-defined classes of behavioral stereotypes, from the movement of a limb to decision making and emotions (Blakemore and Robbins, 2012; Koelsch, 2014; Uhlmann et al., 2017), based on psychological taxonomy or descriptive representations of observable behavioral outputs which are individual- and context-dependent. In these kinds of studies, behaviors are classified into schemes that are either coarse-grained or intuitively defined and biased by human observers’ assumptions (Berman, 2018). Such behavior classifications do not necessarily correspond to inherent behavior structure constrained by biophysics and neural activity, and they preclude the identification of intrinsic neural mechanisms that give rise to behavior—the output of the functioning brain as an integrated system.

Automated behavior quantification and classification using techniques such as machine vision and learning to extract representations of stereotyped behaviors are the first steps towards objectivity and consistency in behavior classification and have the potential to reveal behavioral patterns overlooked by human observers, although these approaches are still based on assumptions and biased (Hong et al., 2015; Robie et al., 2017; Todd et al., 2017; Berman, 2018).

Dense functional mapping is producing huge amounts of data, ranging from molecular and cellular interactions to the connectivity between brain regions and behavioral outputs. Network-based approaches propose to analyze these big, complex data and to model brain networks with theoretical and computational methods such as graph theory and algebraic topology, through statistical inference and dimensionality reduction (Bassett and Sporns, 2017). Although these approaches have the potential to uncover structural and functional features of brain activity, they are subject to methodological and interpretational limitations that result from uncertainties in data acquisition and network definition, thus requiring sophisticated, neurobiologically based brain models down to the molecular scale to reveal the mechanisms underlying brain function and behavior (Sporns, 2014; Medaglia et al., 2015; Bassett and Sporns, 2017).

Organism-level behavior emerges from the interaction of structural connectivity and signaling processes at the molecular, cellular and circuit levels, involving the dynamic activity of huge numbers of molecules and cells as well as multiple physiological and biochemical systems. It is the output of the functioning brain as an integrated system. How can we avoid assumptions in behavior classification that bias our research on the causal relationship between brain structure and function? How can we overcome the barriers of scale and complexity to trace the causal chains of events leading from molecular and cellular mechanisms to brain function and behavior?

### Simulation Neuroscience: From the Squid Giant Axon to the Human Brain

Over past millennia, brain research has evolved through philosophical, experimental and theoretical phases, all of which have contributed to the development of modern neuroscience. Great achievements have been realized in neuronal mapping, connectivity mapping and functional mapping, but these endeavors are hindered by the barriers of scale and complexity. How can we scale up cellular phenotyping and deal with the dynamics of cellular properties to achieve a reliable neuronal cell-type classification? How can we rise to the challenge of volume, time and dynamics in full connectome mapping? How can we identify the molecular and cellular mechanisms that give rise to brain function and behavior? To overcome these fundamental barriers, brain research has to shift to a new phase.

**Figure 3:**
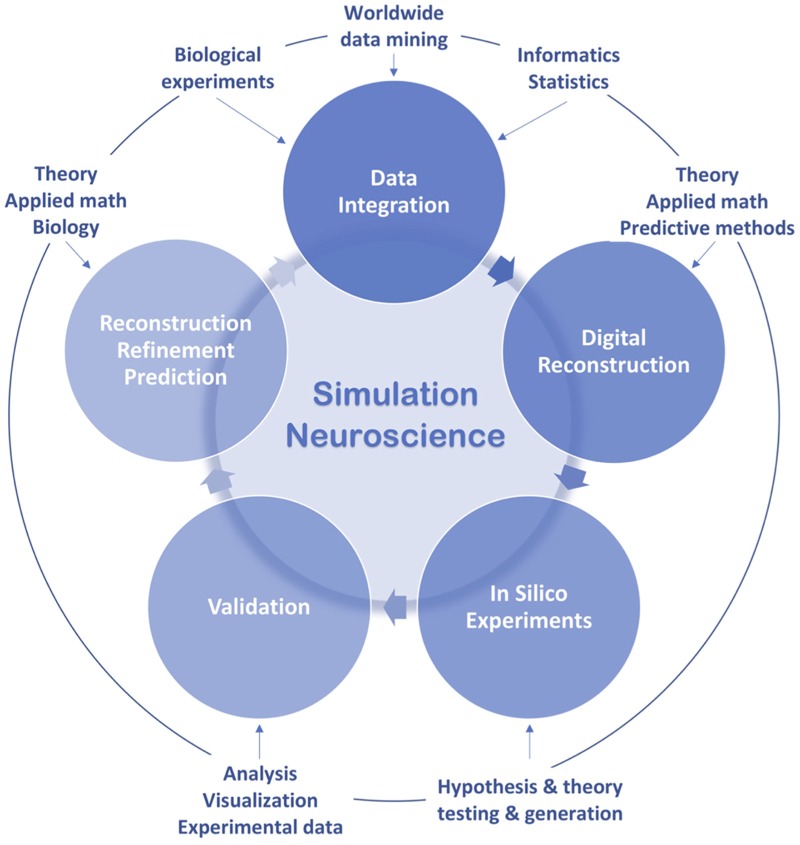
Simulation neuroscience workflow.

Simulation neuroscience aims to fill the gaps in our knowledge of brain structure and function through building a digital copy of the brain with predictive methods, by combining experimental and theoretical approaches (Markram, 2006; Markram et al., 2015; Figure 3). It has the potential to overcome the challenge of scale and complexity. The following pages are aimed at exploring the historical roots of this endeavor by identifying the major milestones that are the most related to it and that are capable of characterizing it in a concise way, instead of conducting an exhaustive survey of all the investigators whose important work has contributed to the evolution of modeling and simulation in neuroscience.

#### All Began With an Axon

Neuroscience originated in a nerve, while detailed simulation in neuroscience began with an axon.

Action potentials were already measured in frog nerve-muscle preparations more than 170 years ago (du Bois-Reymond, 1843), but how is the action potential generated? Although a mathematical model of nerve excitability, the “integrate and fire” model, was built in the early 20th century, based on data obtained from frog nerve stimulation, it was a simple capacitor circuit model (Lapicque, 1907). Since the first measurement of action potentials, the molecular mechanisms of action potential generation had remained an open question over the following 100 years.

More than 60 years ago, two neuroscientists managed to insert voltage clamp electrodes into a squid giant axon and measured the flow of electric current through its surface membrane (Hodgkin and Huxley, 1952). On the basis of their experimental data and inspired by cable theory rooted in the 19th-century model of signaling through submarine telegraph cables (Thomson, 1857), they built a mathematical model of ionic currents to quantitatively account for conduction and excitation and simulated the action potential on the Cambridge University computer. Simulations showed how potassium and sodium ion channels could generate the action potential and predicted the electrical behavior of the axon consistent with experimental data. This was the first detailed digital simulation of a physiological property of a neuron.

Cable theory was further developed to take account of dendritic branching that largely affects neuronal processing. This endeavor gave birth to the first multicompartment dendritic neuron model, based on anatomical and electrophysiological data and simulated on an IBM 650 computer (Rall, 1959, 1962; Segev and Rall, 1998), which was further developed in the following years to unravel the role of dendrites in information transmission (Segev and London, 2000). Single neuron models then evolved into neurocircuit models to study the activity of neuronal populations and synaptic connectivity. The pioneering studies consisted in reconstructions of field potentials and dendrodendritic synaptic circuits in the olfactory bulb for interpreting their underlying mechanisms, based on known anatomical organization and nerve membrane properties and simulated on Honeywell 800 and IBM 370/168 computers (Rall and Shepherd, 1968; Shepherd and Brayton, 1979).

The development of supercomputers in the 1980s drove large-scale simulation of detailed neuron networks, which made it possible to study collective neuronal activities and the neural mechanisms underlying certain brain functions. In 1982, a network model of 100 multicompartment hippocampal neurons, each capable of intrinsic bursting and interconnected by excitatory chemical synapses, was simulated on an IBM 370/168 to reproduce field potentials and intracellular recordings during interictal spikes in epilepsy and to identify the mechanisms underlying this form of neuronal synchronization (Traub and Wong, 1982; IBM Archives, 2003). Six years later, a network of 990 multicompartment hippocampal neurons with different types of cellular interactions was simulated on an IBM 3090 to analyze in particular the mechanisms regulating neuronal synchronization in epilepsy (Traub et al., 1988). At the same time, began the early efforts to simulate neurocircuitry underlying vertebrate behavior, in particular simulation of a segmental network of inhibitory and excitatory interneurons underlying locomotor behavior in lamprey, using Rall neuron models with one soma and a three-compartment dendritic tree, which unraveled the cellular bases of segmental pattern generation, including central and sensory mechanisms and the immediate supraspinal mechanisms initiating locomotion (Grillner et al., 1988, 1991).

In the early 1990s, the simulator “NEURON” was developed for empirically based simulations of single and networks of neurons with complex anatomical and biophysical properties, such as complex branching morphology, multiple channel types, inhomogeneous channel distribution, ionic diffusion and the effects of second messengers (Hines, 1989, 1993). During the same period, was released the GEneral NEural SImulation System (GENESIS), a simulation environment for constructing realistic models of neurobiological systems from subcellular processes and individual neurons to networks of neurons and neuronal systems (Wilson et al., 1989; Bower et al., 2013). In the following years, simulators such as MCell and STEPS were developed to simulate biochemical signaling pathways at the molecular scale (Stiles et al., 1996; Hepburn et al., 2012). As detailed models of neural systems have become more and more sophisticated, efforts are underway to develop a language that provides a common data format for defining and exchanging descriptions of these detailed models, such as the NeuroML project which aims to develop an eXtensible Markup Language (XML) based description language (Goddard et al., 2001).

In parallel with the development of simulators, large-scale simulations continued to grow. A single-column thalamocortical network model with 3,560 multicompartment neurons, including seven cell types characterized by different types of morphology, connectivity and electrical behavior, was simulated on a Linux cluster (IBM e1350) to particularly address the physiology of network oscillations and epileptogenesis (Traub et al., 2005). Although the model exhibited gamma oscillations, sleep spindles and epileptogenic bursts, it was insufficient to describe other neuronal network behaviors, particularly due to the omission of many cell types, many unknown structural details, the absence of synaptic plasticity and the restriction of the model to a single column. In the modelers’ view, their work represented an extremely preliminary step towards understanding subtle aspects of brain function, such as learning or information processing, and they hoped for more detailed models to study a broader range of network phenomena. They considered that detailed modeling of extensive brain circuits was necessary for understanding brain function and for making important experimental predictions that would not have been made without the model.

These previous endeavors mainly aimed to build models to reproduce certain brain functions or dysfunctions, such as action potential generation or neuronal synchronization in epilepsy. However, to trace the causal chains of events leading from molecular and cellular mechanisms to diverse brain functions and behaviors, biologically realistic dense reconstructions of the brain realized without the goal of fitting the model to any specific function (if reconstructions are correct, functions should arise naturally) are demanded. This need led to the birth of simulation neuroscience in the early 21st century (Markram, 2006). Since then, digital reconstructions have increased in size and biological accuracy to unravel deeper mechanisms underlying brain function. To date, the most detailed reconstruction concerns the microcircuitry of rat somatosensory cortex, containing ~31,000 multicompartmental conductance-based neuron models, including 55 layer-specific morphological and 207 morphoelectrical subtypes, and simulated on supercomputers such as the Blue Brain IV, ranked the 100th most powerful supercomputing system (Top500, June 2015). This digital reconstruction is able to generate emergent network activity and to reproduce an array of *in vitro* and *in vivo* experiments without parameter tuning, and it enables experiments so far impossible either *in vitro* or *in vivo* (Markram et al., 2015).

Since its origin, detailed simulation in neuroscience has evolved from a single cell type to more than 200 cell types characterized by morphological and physiological features, from one type of synaptic connectivity to the predicted anatomical and physiological properties of all the intrinsic synapses formed onto and by any neuron, from specific models aimed at reproducing certain forms of neuronal activity to generic dense reconstructions of brain regions with various neuronal activity patterns and emergent network behaviors, from an action potential generated through a squid giant axon to diverse network behaviors of rat neocortical microcircuitry with 31,000 neurons connected through 36 million synapses. A large body of disconnected experimental datasets and knowledge accumulated since the origin of neuroscience have been integrated into a unified digital copy of neocortical microcircuitry, allowing deeper insights into the neural mechanisms underlying brain function. Efforts are underway to reconstruct more electrophysiological and biochemical mechanisms and to simulate the human brain.

#### Transcending Scale and Complexity

**Figure 4:**
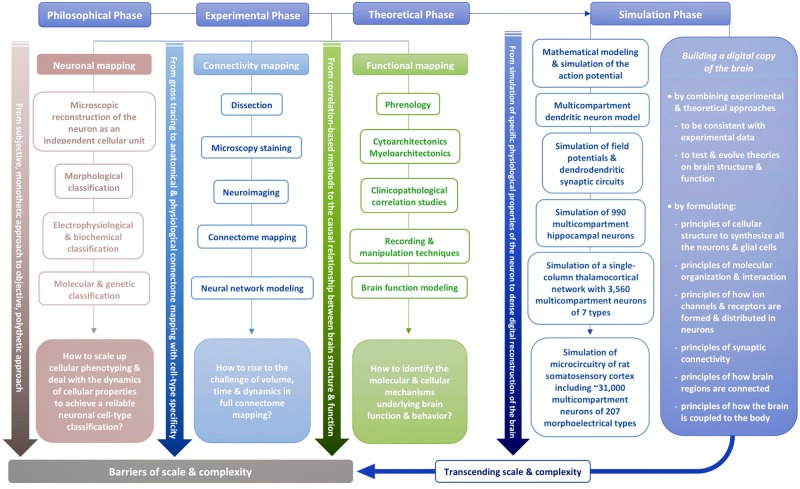
Transcending scale and complexity.

Simulation neuroscience identifies strategic data and formulates principles of brain structure and function to accelerate our understanding of the brain, instead of experimentally mapping all the elements and activities in the brain, which is impossible to achieve due to their scale and complexity (Markram, 2006; Markram et al., 2015; Figure 4).

##### Neuronal Reconstruction and Simulation

Reconstructions of single neurons are the building blocks of the digital brain. In the early years of simulation in neuroscience, some researchers were aware of the importance of describing the detailed structure of neurons to simulate the voltage response to inputs impinging on the cell in different locations and interactions between cells generated by extracellular current flows. They were also aware of the importance of reconstructing the diverse types of electrical behavior of neurons. Therefore, they argued against using point neuron models (Traub et al., 1988). Nevertheless, at this stage, the endeavor to digitally reconstruct the morphological and physiological types of neurons was limited in scale and accuracy, so new approaches were to be developed.

Historically, neuronal morphologies were first qualitatively described through visual inspection, then quantitatively described based on morphometric parameters. Since these methods are not standardized to objectively describe complex branching patterns of neuronal trees, topological methods have been developed in simulation neuroscience to rigorously quantify the structural differences of neuronal trees and to classify neurons into distinct morphological types by encoding the spatial structure of each neuronal tree with a unique topological signature (Kanari et al., 2018). Then cloning each morphological type with statistical variations allows scaling up the reconstruction of neurons belonging to each morphological type while respecting biological variability.

Automated statistical analysis can reveal distinctive electrical types; computational multi-parametric approach can extract combinatorial expression rules of ion channel genes underlying electrical phenotypes; ion channels can be automatically inserted by simulators combined with an automated fitting algorithm. These methods developed in simulation neuroscience allow objective and high-throughput reconstruction of electrical types (Khazen et al., 2012; Druckmann et al., 2013; Markram et al., 2015).

The high-throughput digital reconstruction of different types of neurons can be extended from morphological and electrophysiological features to other dimensions such as projection and molecular features when sufficient data that allow quantifying these features become available. Furthermore, the structure and function of brain cells vary according to their position in the brain; this should be considered while reconstructing different classes of brain cells. To support this endeavor, whole-brain cell atlases are being built, providing insights into cellular organization only possible at the whole-brain scale. The first dynamic 3D cell atlas for the whole mouse brain has recently been achieved, showing cell positions constructed algorithmically from whole brain Nissl and gene expression stains, and providing the densities and positions of all excitatory and inhibitory neurons, astrocytes, oligodendrocytes and microglia in each of the 737 brain regions defined in the Allen Mouse Brain Atlas (Erö et al., 2018).

During the evolution of simulation neuroscience, the digital reconstruction of different types of neurons has become more and more multi-constrained, realistic and high-throughput, and it allows evolving current neuronal classifications (Deitcher et al., 2017). Today, we have objective classification of morphologies which is helping define morphological types; we have more or less agreed electrical protocols that can be used to describe electrical types; we have tracing studies that are helping define the projection types, and we have single cell transcriptome data that are beginning to describe the genetically different types of cells. Efforts are underway to define a minimum sample size capable of reliably revealing distinct types of brain cells, to reduce dimensionality by defining a relevant level of granularity and to identify permanent molecular features that maintain cell identity—a step forward towards an objective and comprehensive classification of neuronal types.

##### Connectivity Reconstruction and Simulation

More than 100 years ago, Santiago Ramón y Cajal initiated predictive reconstruction by inferring neuronal connectivity from morphological features of neuronal arbors (Ramón y Cajal, 1894). About 80 years later, trying to digitally reconstruct neuronal circuits, some researchers considered pointless to explicitly specify all the neuronal connections, which is unattainable experimentally (Traub et al., 1988). They chose to reconstruct neuronal connections by a series of random choices, based on the statistical properties of the network topology, such as the average number of inputs or outputs per cell and the probability of connection between pairs of cells.

New approaches based on statistical modeling and synaptic rules have been developed in simulation neuroscience to accurately predict synaptic connectivity (Perin et al., 2011; Hill et al., 2012; Ramaswamy et al., 2012), in particular data-driven algorithmic approaches based on established principles of synaptic connectivity and constrained by interdependencies between microcircuit properties such as the number of synapses and bouton densities. With these approaches, it is possible to predict the number and location of all synaptic connection types shown experimentally and connection properties impossible to measure experimentally such as the number of source and target cells and synapses (Markram et al., 2015; Reimann et al., 2015). The physiology of synapses can be predicted by formulating rules of synaptic types based on experimental data to generate a relatively complete map of synaptic dynamics (Markram et al., 2015). Synaptic plasticity rules can also be formulated (Kalisman et al., 2005; Loebel et al., 2013). In this way, it is possible to predict the anatomical and physiological properties of all the intrinsic synapses formed onto and by any neuron. These predictions combined with future experiments could be used to further refine connectivity reconstruction and simulation.

##### Functional Reconstruction and Simulation

More than 60 years ago, Hodgkin and Huxley’s reconstruction and simulation of the action potential predicted the properties of the gating structures of ion channels (Hodgkin and Huxley, 1952), showing the power of simulation in neuroscience to unravel biological mechanisms long before their experimental observations. Since the birth of simulation neuroscience, digital reconstructions and simulations have been used to fill the vast gaps in our data, to interpret experimental observations and identify the underlying mechanisms, and to test and generate theories about brain function and dysfunction (Markram, 2006; D’Angelo, 2014; Frackowiak and Markram, 2015).

To identify neural mechanisms that give rise to emergent complex behavior, reconstructing and simulating neurons embedded in microcircuits, microcircuits embedded in brain regions and brain regions embedded in the whole brain is an approach consistent with the biological reality that organism-level behavior is the output of the functioning brain as an integrated system, from molecular and cellular interactions to connections between neurocircuits and between brain regions. Neurorobotics, combined with digital reconstructions, creates new possibilities for studying neural mechanisms leading to emergent behavior across different spatiotemporal scales (Falotico et al., 2017).

The deep relationship between structure and function that guided the first investigators at the origin of neuroscience is the foundation of simulation neuroscience. Recent digital reconstructions and simulations of rat neocortical microcircuitry could reproduce the spatial mode and the temporal dynamics of empirically observed functional networks without parameter tuning and showed emergent network states modulated by physiological mechanisms (Markram et al., 2015). In the same reconstructions, a new algebraic topology approach revealed that synaptic networks contain an abundance of cliques of neurons bound into cavities that guide the emergence of correlated activity, showing a formal link between neural network structure and function (Reimann et al., 2017b). Our understanding of brain structure and function is being deepened through building a digital copy of the brain.

##### Perspectives and Challenges

The dense digital reconstruction of the brain from sparse, complementary datasets by predicting biological parameters that are not available experimentally involves dealing with the relationships between known and unknown parameters, deriving principles from experimental data, and reducing biological complexity while preserving the principles of brain structure and function.

Initial digital reconstructions need to integrate more types of neural mechanisms and signaling systems, such as neuro-glio-vascular unit and neuromodulation (Jolivet et al., 2015; Ramaswamy and Markram, 2018). They will be challenged and refined by new experimental observations. As more types of datasets and parameters are to be integrated, more relevant biological principles have to be derived, and programming complexity will largely increase. Efficient computational methods have to be developed to satisfy the requirements of this nascent science in rapid evolution. Simulation neuroscience is rising to these challenges and constitutes an essential phase of brain research towards transcending scale and complexity to causally link molecules, genes and cells to brain function and behavior.

#### The Next Phase of Brain Research

Simulation neuroscience is an efficient approach to integrating disconnected datasets and knowledge in neuroscience that have been accumulated over hundreds of years. The extraction of the rules of the relationships between datasets that concern different levels of brain organization helps to build an integrated view of brain structure and function (Tiesinga et al., 2015). Through digital reconstructions and simulations, researchers can conduct in silico experiments, improve experimental methods, test and generate hypotheses and theories, make predictions and suggest new experiments (Druckmann et al., 2011; Reimann et al., 2013; Abdellah et al., 2015; Hay and Segev, 2015).

Neuromorphic computing uses very-large-scale integration (VLSI) systems containing electronic analog circuits to mimic neuroarchitectures of the nervous system (Mead, 1990). This approach has the potential to overcome the major limitations of traditional computing, such as energy consumption, software complexity and component reliability. Current neuromorphic computing consists in large-scale simulations of neuronal connectivity with few biological details (Furber et al., 2014; The FACETS Project, 2018). This research field would benefit from simulation neuroscience, which has the potential to provide the blueprints of neurocircuits.

Without deeper insights into the fundamental mechanisms underlying brain function, we cannot effectively treat neurological disorders, which result from dysfunctions of neural systems down to the molecular scale. The widely known neurodegenerative disease, Alzheimer’s disease, was described more than 110 years ago (Alzheimer, 1906). Today, there is still no effective treatment (The Lancet, 2016). In fact, this “disease” is poorly defined, referring to an array of various symptoms ranging from memory loss to diverse cognitive impairments, caused by multiple distinct brain dysfunctions (Scheltens et al., 2016; Frisoni et al., 2017). How can we treat a brain disease if we cannot identify its underlying mechanisms and clearly define it? How can we restore brain dysfunctions if we do not even understand the neural mechanisms underlying normal brain function? Deep understanding of brain structure and function is fundamental to clinical research, which will make it possible to identify the “biological signature” of each brain dysfunction instead of simple syndromic descriptions (Frackowiak and Markram, 2015). This is why biologically realistic digital reconstructions of the brain can be a valuable tool for modeling and simulating brain dysfunctions and for developing and validating treatments (D’Angelo, 2014; Frackowiak and Markram, 2015).

Our understanding of brain structure and function is being deepened as we build and refine a digital copy of the brain. Each step unravels new aspects of brain structure and function in a systematic manner. Even though an accurate and complete reconstruction and simulation of the human brain will require at least yottaflop (10^24^ flops) computing or even more^1^, we are getting closer to a comprehensive understanding of the brain by developing multiscale simulations. According to the nature of the studied question, some parts of the brain can be simulated at low resolution, and others at high resolution. This allows accelerating our understanding of the brain even before enough computing power becomes available. Finally, it would be possible to trace the neural mechanisms leading to the emergence of biological intelligence and to challenge the foundations of our understanding of consciousness through building a digital copy of the brain.

## Understanding the Multiscale Brain

Since the dawn of neuroscience, hundreds of years ago, this human endeavor has fundamentally been a series of reconstructions: reconstruction of the neuron as a single cellular unit; reconstruction of neurons into distinct types according to their morphological, electrophysiological, biochemical and molecular properties; reconstruction of neural connectivity between brain regions, neuronal groups, individual neurons; reconstruction of the neural mechanisms underlying brain function and behavior. In attempting to complete the reconstruction of brain structure and function, experimental and theoretical approaches are hindered by the fundamental barriers of scale and complexity.

**Figure 5:**
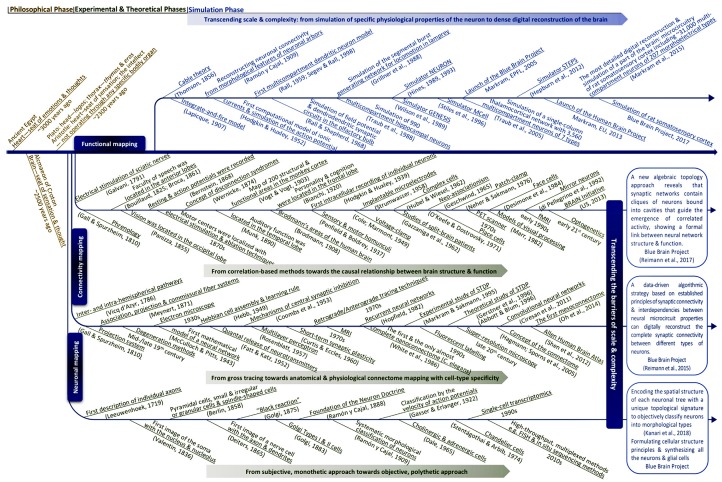
Evolutionary milestones towards simulation neuroscience.

To overcome these barriers, the tools for reconstructing neurons and the brain have dramatically evolved, from Leeuwenhoek’s self-made one-lens microscope to compound achromatic microscope and Ramón y Cajal’s pencil until today’s supercomputers. Leveraging high performance computing, data analysis and statistical inference methods as well as algorithmic approaches, simulation neuroscience quantifies, integrates, scales up and accelerates all the previous reconstruction processes and evolves them into a unified digital copy of the brain—a quantitative and qualitative shift through the dense digital reconstruction and simulation of the brain from sparse experimental data, with the aim of causally linking molecular, cellular and synaptic interactions to brain function and behavior (Figure 5).

Since the introduction of the first supercomputers in the mid-20th century, in 70 years, processing power has increased from ~10^3^ to ~143.5 × 10^15^ flops (Dongarra, 2006; November, 2018 | TOP500 Supercomputer Sites). Since the first observation of nerve fibers, the microscopic and physiological reconstruction of the neuron as an independent cellular unit had taken almost 240 years, while the evolution from the first digital reconstruction of the action potential to the dense digital reconstruction of neocortical microcircuitry took about 60 years. What will the future hold for the reconstruction and simulation of the entire brain?

From the dawn of human civilization, the advances in brain research have been generated through a series of fundamental shifts in the types of human thinking to understand the mind and the brain. Relying on intuitive and analogical thinking, ancient philosophers tried to address fundamental questions but were unable to provide empirical evidence. Seeking evidence, reductionist thinkers in experimental neuroscience have gained a deep understanding of many components of the brain but have also produced a huge number of disconnected datasets and knowledge. Theoretical neuroscience applies abstractive thinking to be free from the details in the brain, which may advance artificial intelligence but leaves open the question whether it will advance our understanding of the causal links between brain structure and function. To transcend these barriers, brain research needs a new way of thinking and a new approach. This new phase is proposed to be simulation neuroscience, which is based on integrative and predictive thinking.

Will simulation neuroscience be able to go deep enough through multiple different layers to finally understand the multiscale brain, to answer the probably ultimate question for us, humans, of understanding ourselves, which has haunted us since the dawn of time?

Atoms are combined into molecules; DNA molecules are bound into sequences to produce genes; genes produce proteins; different combinations of proteins produce various types of cells, which are combined into different brain regions to finally form the unique human brain. How do these complex mechanisms interact, leading from single atoms and molecules to brain function and behavior? How does the brain create our small world immersed in the universe? How does the brain incorporate our experiences that define our existence? Still so many unsolved questions.

After thousands of years of brain research, hundreds of years of neuroscience, we remain strangers to ourselves. From the Temple of Apollo, traveling through millennia, the Delphic maxim is still resonating: “Know thyself (Pausanias, 1918).” What will the future hold for us, in 10 years, 100 years, 1,000 years? To understand the multiscale brain, neuroscience now has to shift to a new phase.

## Author Contributions

XF and HM conceived the research and wrote the text. XF wrote most of the text and made all the figures.

## Conflict of Interest Statement

The authors declare that the research was conducted in the absence of any commercial or financial relationships that could be construed as a potential conflict of interest.

## Footnotes

^1^High-resolution real-time molecular simulation of the human brain would need ~4 × 10^29^ flops*: • ~90 billion neurons, ~1,000 trillion synapses • ~90 billion glial cells, ~450 billion vascular end feet, supporting ~450 trillion synapses • ~1 trillion molecules/cell, ~1,000 reactions/molecule/s, ~20 diffusion jumps/s • ~10,000 time steps/s *Henry Markram. “Will Computers Become as Capable as the Brain?” presented at the conference “What Makes Us Human: From Genes to Machines,” The Hebrew University, Jerusalem, June 4–6, 2018.
